# Plasticity and ductility in graphene oxide through a mechanochemically induced damage tolerance mechanism

**DOI:** 10.1038/ncomms9029

**Published:** 2015-08-20

**Authors:** Xiaoding Wei, Lily Mao, Rafael A. Soler-Crespo, Jeffrey T. Paci, Jiaxing Huang, SonBinh T. Nguyen, Horacio D. Espinosa

**Affiliations:** 1 Department of Mechanical Engineering, Northwestern University, 2145 Sheridan Road, Evanston, Illinois 60208, USA; 2 Theoretical and Applied Mechanics Program, Northwestern University, 2145 Sheridan Road, Evanston, Illinois 60208, USA; 3 Department of Chemistry, Northwestern University, 2145 Sheridan Road, Evanston, Illinois 60208, USA; 4 Department of Chemistry, University of Victoria, British Columbia, V8W 3V6, Canada; 5 Department of Materials Science and Engineering, Northwestern University, Evanston, Illinois, 60208, USA

## Abstract

The ability to bias chemical reaction pathways is a fundamental goal for chemists and material scientists to produce innovative materials. Recently, two-dimensional materials have emerged as potential platforms for exploring novel mechanically activated chemical reactions. Here we report a mechanochemical phenomenon in graphene oxide membranes, covalent epoxide-to-ether functional group transformations that deviate from epoxide ring-opening reactions, discovered through nanomechanical experiments and density functional-based tight binding calculations. These mechanochemical transformations in a two-dimensional system are directionally dependent, and confer pronounced plasticity and damage tolerance to graphene oxide monolayers. Additional experiments on chemically modified graphene oxide membranes, with ring-opened epoxide groups, verify this unique deformation mechanism. These studies establish graphene oxide as a two-dimensional building block with highly tuneable mechanical properties for the design of high-performance nanocomposites, and stimulate the discovery of new bond-selective chemical transformations in two-dimensional materials.

## Abstract (web summary)

Biasing chemical reaction pathways in a particular molecule may lead to new material properties. Here, the authors report mechanochemical covalent epoxide-to-ether functional group transformations, deviating from classical epoxide ring-opening reactions, in suspended graphene oxide membranes.

## Graphical abstract

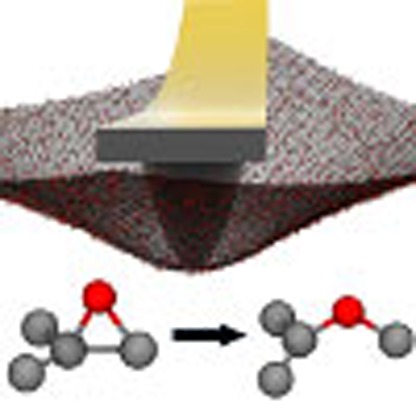

While single atomic layers with large lateral dimensions, such as graphene-based sheets, have attracted significant attention for their high strengths and elastic moduli^1^, their potential as a platform for exploring novel chemical transformations with nanoscale mechanical means has been unexplored. In theory, the combination of large area, tuneable chemical functionality and mechanical robustness should make these materials excellent complements to DNAs and proteins for exploring covalent bond-selective chemistry in two dimensions, extending our current knowledge of mechanically induced chemical transformations beyond these biopolymers, and hydrogen bonding and van der Waals interactions^2^,^3^. In particular, graphene oxide (GO), an oxidized derivative of graphene, offers a tremendous opportunity for directly probing how the supporting chemical functionalities on its basal plane, such as epoxide and hydroxyl groups, respond to mechanical perturbations at the atomic level. For example, these various oxygenated functional groups, which are traditionally viewed as defect sites in the *sp*^2^ network of the parent graphene sheet and deemed to have less robust mechanical properties^4^,^5^,^6^, would be more likely to respond to activation by mechanical forces without undergoing catastrophic failures.

Herein, we report that the chemical functionalities on GO can indeed confer damage-tolerant deformation mechanisms and mechanical properties that are unattainable in graphene. While defect-free graphene exhibits brittle failure, the cyclic epoxide groups on GO help to dissipate strain energy and hinder crack propagation through a novel epoxide-to-ether transformation, making it ductile. This chemically induced plasticity in GO is verified through membrane deflection experiments and rationalized by density functional-based tight-binding (DFTB) calculations. These findings reveal a unique relationship between the chemical structures and mechanical properties of GO at the atomic level, and demonstrate an example of mechanically activated, covalent bond-selective chemistry of the epoxide groups that completely differ from its classical type of ring-opening reactions.

## Results

### Mechanical characterization

**Figure 1:**
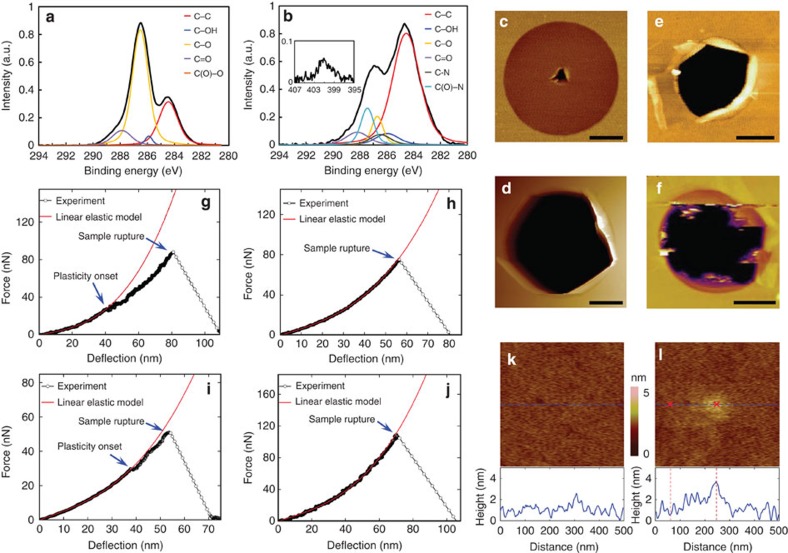
Characterization of Graphene oxide and amine-modified GO. (**a**,**b**) C1s XPS spectra for GO (**a**) and A-GO (**b**), respectively. (**b**, inset) N1s XPS spectrum for A-GO. (**c**,**d**) AFM topology images of ruptured monolayer GO (**c**) and A-GO (**d**) membranes after membrane deflection tests, respectively. (**e**,**f**) AFM topology images of ruptured pristine graphene and less-oxidized GO, respectively, after membrane deflection tests (images were adapted from refs 1, 6). (**g**–**j**) typical ductile and brittle force versus deflection curves for suspended GO (**g**,**h**) and A-GO (**i**,**j**) membranes, respectively. (**k**,**l**) AFM scanning images of a 500 × 500 nm area at a suspended GO membrane centre before (**k**) and after (**l**) testing. Scale bar, 500 nm (**c**,**d**,**e**). Scale bar, 1 μm (**f**).

The GO nanosheets used in this work were synthesized using a modified Hummers method^7^, and are extensively functionalized with epoxide groups based on X-ray photoelectron spectroscopy (XPS) analysis (Fig. 1a; see also Supplementary Table 1). To further confirm the epoxide-rich composition, we compared the C1s XPS spectrum of our GO to two other previously reported materials with well-characterized composition: highly oxidized GO with predominantly epoxide groups^7^, and less-oxidized GO with predominantly hydroxyl groups^6^ (see Supplementary Note 1). We found the composition and C1s XPS spectrum of our GO to be very similar to those of the epoxide-rich material, and markedly different from those of the epoxide-poor material (see Supplementary Table 2 and Supplementary Fig. 1). By Langmuir–Blodgett deposition^8^, these sheets were first deposited over an array of circular microwells that were prefabricated on a silicon substrate (see Supplementary Figs 2 and 3). The centre of each individual circular membrane was then deflected with an atomic force microscopy (AFM) diamond probe to measure mechanical properties (see Supplementary Fig. 4). Figure 1g,h shows the two types of force versus deflection responses for suspended GO membranes that correspond to ductile and brittle failure modes, respectively. In the ductile failure mode, the force versus deflection response can only be fit to a linear elastic membrane solution (Equation 2) during the initial stage of deflection, beyond which (∼40 nm) the suspended GO monolayer deformed inelastically until rupture. In contrast, the linear elastic behaviour is observed throughout the deflection in the brittle failure mode. At the peak force, an abrupt increase in deflection occurred, indicating sudden film rupture.

Notably, only 1 suspended GO monolayer among the 19 that we tested showed brittle failure, most likely due to the occasional large (that is, >10 nm) defects in the basal plane of the GO nanosheet, which comprises relatively uniform, randomly distributed nanometer-sized domains of graphitic and oxygenated carbon atoms^9^,^10^,^11^. A typical AFM image of the ruptured ductile GO monolayer (Fig. 1c) clearly shows a localized puncture at the centre, which is in remarkable contrast to the catastrophic rupture of pristine graphene or less-oxidized GO containing mainly hydroxyl groups (Fig. 1e,f)^1^,^6^. The radius of the tear was ∼150 nm, which is consistent with the dimension of the tip cross-section at maximum penetration, suggesting the presence of a unique crack-arresting mechanism in GO. This is confirmed in a second set of experiments, where the AFM tip was retracted quickly after reaching the maximum load but before membrane rupture. AFM images of these GO membranes before and after deflection clearly show a ‘damage' zone at the membrane centre (Fig. 1k,l), ∼100 nm in diameter and 1–2 nm higher than the undamaged region, where the material underwent a severe plastic deformation.

### Theoretical analysis

**Figure 2:**
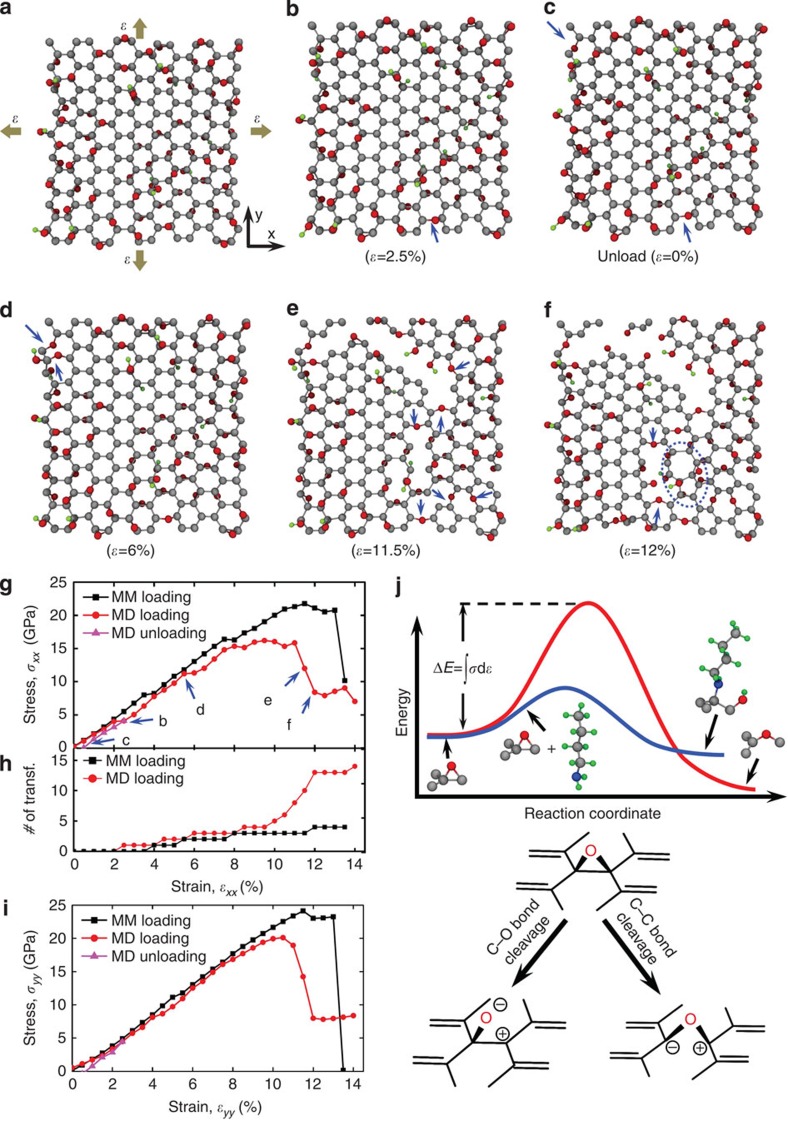
Density functional-based tight-binding modelling. Modelling of a 1.988 × 2.091 nm^2^ GO sheet (*ϕ*=0.7) being subjected to equibiaxial tension (**a**–**f**). (**a**) The initial configuration of the GO sheet and the schematic of the constraints. Grey, red and green beads represent carbon, oxygen and hydrogen atoms, respectively. (**b**–**f**) The snapshots of the deformed GO sheet during molecular dynamics (MD) simulations. The dark-blue arrows highlight the locations on each snapshot where epoxide-to-ether transformations occurred. The dashed circle in snapshot IV highlights a Stone–Wales defect. (**g**) Stress–strain curves in the armchair direction (*x* axis in **a**) obtained from molecular mechanics and MD simulations. Labels in stress–strain curve refer to MD snapshot panels in this figure. (**h**) Accumulated number of epoxide-to-ether transformations as a function of strain. (**i**) Stress–strain curves along the zigzag direction (*y* axis in **a**). (**j**) An illustration of the relative energetic difference between the mechanochemically induced epoxide-to-ether transformation activated by strain energy (that is, C–C bond cleavage, red profile) and the epoxide ring-opening by *n-*butylamine (that is, C–O bond cleavage, blue profile). Grey, red, green and blue beads represent carbon, oxygen, hydrogen and nitrogen atoms, respectively. The chemical drawings beneath the profiles are included only to illustrate the key differences between the two chemical pathways without including all the relevant species (water, *n-*butylamine and so on) that can be involved to facilitate the transformations. As such, the formal charges that are indicated on the drawings should not be taken literally.

To explore the origin of the experimentally observed plasticity, we modelled the tensioning of graphene and GO through a series of semi-empirical DFTB calculations using the open-source code CP2K (http://www.cp2k.org/). We first generated a molecular model of ∼2 × 2 nm^2^ GO sheet with a functionalization level *ϕ*=0.7 (defined as the fraction of oxidized carbon atoms). A 4:1 epoxide-to-hydroxyl functional group ratio was used to resemble the epoxide-rich composition confirmed by XPS analysis. A Monte Carlo-based Rosenbluth sampling algorithm was employed to determine the favourable locations of the functional groups from random choices according to a Boltzmann-like distribution (see the Methods section for the algorithm implementation). The obtained model shows excellent agreement with structural features previously reported for theoretically studied GO sheets in the literature (see Supplementary Notes for further details)^12^,^13^,^14^,^15^,^16^,^17^. Then, we carried out molecular mechanics and molecular dynamics calculations to investigate the plasticity mechanism by applying equibiaxial tension on the nanosheet (Fig. 2a), similar to the constraint on the material during membrane deflection experiments.

As shown in Fig. 2g, the stress–strain response of the GO nanosheet along the armchair direction shows strain bursts at 3.5% strain in molecular mechanics (and 2% strain in molecular dynamics) simulations that appear to correspond with a mechanochemical epoxide-to-ether transformation reaction (Fig. 2b). This reaction, biased by strain energy (Fig. 2j), activated at stress levels of 8.0 GPa in molecular mechanics and 4.0 GPa in molecular dynamics simulations, respectively. The lower stress obtained from molecular dynamics (at 300 K) in comparison with molecular mechanics (at 0 K) suggests that this strain-energy-activated mechanochemical transformation is more favourable at ambient temperature, where the experiments were carried out. The ether groups that formed remained after unloading from 2.5% strain to 0% strain in our molecular mechanics simulation (Fig. 2c), confirming that this reaction is irreversible and deformation is plastic. In a previous report, using density functional theory (DFT), Li *et al.* studied graphene and carbon nanotube unzipping during oxidative processes^18^. They showed that a spontaneous epoxide-to-ether transformation would happen if multiple epoxide groups align on the opposite ends of benzene rings in the same side of the graphitic basal plane to form a linear fault line. However, this particular configuration of linearly aligned epoxy groups considered by Li *et al.* is only a transient state (that is, highly unstable), and is statistically unlikely in the case of the stable suspended GO membranes studied herein. The GO models generated in this study using the Monte Carlo algorithm suggest that this fault line of epoxide groups is energetically unfavourable. Rather, our study reveals that epoxide groups in GO are randomly distributed and form a stable structure. The epoxide-to-ether transformation occurs only when the GO sheet is under a substantial mechanical stress (between 4.0 and 8.0 GPa) and leads to improved material toughness. Thus, considering these essential distinctions, the scenarios discussed by Li *et al.* and herein are significantly different.

As the strain increased, more epoxide-to-ether transformations accumulated; at 6% strain, a second major strain burst was observed in the molecular dynamics stress–strain curve as the result of the strain energy release at the bond transformation locations (Fig. 2d). At 9.5% strain, a nanoscale crack appeared in our simulation model (Fig. 2e) but did not lead to a catastrophic failure of the material. Rather, it corresponds to a significant number of mechanically induced epoxide-to-ether transformations as the strain was increased (Fig. 2h). The accumulation of these transformations led to a plateau in the stress–strain curve, indicating a delay in crack growth. At the end of this plateau, crack growth led to a stress drop and failure. The transition captured in Fig. 2e,f clearly shows that the epoxide-to-ether transformation at the crack front is responsible for energy dissipation, presumably due to the blunting of the crack front by the higher flexibility offered by the C–O–C angle in the newly formed ether group. At 12% strain, a void initiated near the crack tip, and a Stone–Wales defect, commonly observed during failure in graphitic materials^19^, formed beside the void (Fig. 2f).

As described above, the molecular dynamics stress–strain curve shown in Fig. 2g clearly demonstrates the plasticity and damage tolerance of GO when being tensioned in the armchair direction. In contrast, the corresponding stress–strain curve in the zigzag direction (Fig. 2i) exhibits negligible plastic behaviour, suggesting that the mechanochemical response to strain energy in GO is chirality dependent. Together, these results provide an unexpected explanation for the predominantly ductile failure mechanism in our experiment: as shown by the molecular dynamics snapshots in Fig. 2a–f, the novel epoxide-to-ether transformation that occurs on the basal plane of a GO nanosheet on indentation can readily accommodate a network of nanoscale cracks and prevents it from catastrophic failure until these nanocracks coalesce. This is consistent with the experimental observation of a damage zone in the suspended GO membrane after testing (Fig. 1l).

### Amine modification of GO sheets

Our results thus far suggest that the epoxide-to-ether transformation in the basal plane of GO is the origin of the plasticity and the ductile failure behaviour that we observe in our experiments. Therefore, if the epoxide groups are removed such as through amine-induced ring-opening reactions^20^, GO should show a more pronounced brittle failure behaviour. This is indeed the case: when 18 samples of *n-*butylamine-modified GO (A-GO) were tested, brittle failure was observed much more frequently than in the case of GO. Eight of the samples exhibited clear brittle failure (Fig. 1j), and while the remaining samples showed a ductile failure behaviour, the degree of plastic deformation in them is significantly less than that in the GO membranes discussed earlier (cf. Fig. 1i,g). Furthermore, the typical rupture topology of a suspended monolayer A-GO membrane that exhibited brittle failure (Fig. 1d) showed features that are similar to those in pristine graphene and less-oxidized GO containing mainly hydroxyl groups (Fig. 1e,f)^1^,^6^. Together, these data support our assertion that the presence of epoxide groups, and thus the availability of epoxide-to-ether transformations, is responsible for the plasticity of the original GO samples. Presumably, the ring-opening reactions of the epoxide groups by *n-*butylamine^20^ (see Fig. 1b, Supplementary Table 1 and Supplementary Notes) have rendered A-GO more brittle.

**Figure 3:**
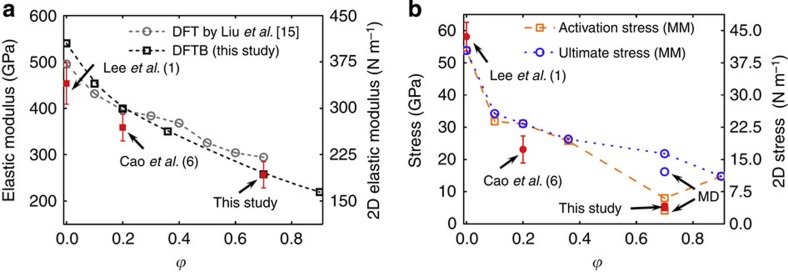
Elastic, plastic and failure analysis of graphene oxide (GO). (**a**) Comparison of elastic moduli predicted by density functional theory (adopted from ref. 15) and density functional-based tight-binding (DFTB) for GO with increasing *ϕ* with experimental results for graphene (that is, *ϕ*=0) from ref. 1, GO with *ϕ*=0.2 from ref. 6, and GO with *ϕ*=0.7 (this study). (**b**) Comparison of ultimate and activation stresses predicted by molecular mechanics with values reported for graphene (three-dimensional stress was converted by assuming a GO thickness of *h*=0.75 nm), GO with *ϕ*=0.2 and GO with *ϕ*=0.7. Molecular dynamics predictions of ultimate and activation stresses for GO with *ϕ*=0.7 are also plotted. Hollow and solid symbols represent DFTB predictions and experimental results, respectively. Error bars in **a** and **b** refer to s.d.'s.

The pre-stress and elastic modulus values of our GO and A-GO, as derived from the elastic analysis of the experimental measurements (see Supplementary Fig. 5 and Supplementary Notes) also support our conclusion. Assuming an effective GO thickness of *h*=0.75 nm (ref. 21), the higher pre-stress in A-GO (0.9±0.2 GPa) compared with that for our original GO with *ϕ*=0.7 (0.65±0.3 GPa) suggests that amine modification did indeed increase membrane tension. We note that the value for our original GO was notably higher than that reported by Cao *et al.*^6^ (0.14±0.02 GPa by assuming the same GO thickness *h*=0.75 nm) with *ϕ*=0.2, presumably due to stronger interactions between the basal planes of our highly oxidized nanosheets and the substrate. In contrast, the elastic modulus of A-GO is 223.3±33.2 GPa, which is slightly lower than that of the original GO with *ϕ*=0.7 (elastic modulus *E*=256.4±28.2 GPa; elastic modulus in two-dimensional (2D) form *E*^2D^=192.3±21.2 N m^−1^) as a result of the ring opening of the epoxide groups. Both of these values are much lower than those reported by Cao *et al.*^6^ (*E*=384±31 GPa, *E*^2D^=269±21 N m^−1^) for a GO sample with *ϕ*=0.2, suggesting that the elastic modulus for GO decreases with increasing levels of functionalization. This conclusion is further supported by the good agreement between our experimental measurements and the predicted elastic properties extracted from additional DFTB calculations on GO nanosheets with various functionalization levels (*ϕ*=0.1, 0.2, 0.36, 0.7 and 0.9) (Fig. 3a; see also Supplementary Fig. 6, Supplementary Table 3 and Supplementary Notes). Furthermore, our DFTB simulations agree very well with DFT predictions by Liu *et al.* for disordered GO models at the same functionalization levels despite differences in functional group ratios (a 1:2 epoxide-to-hydroxyl group ratio was used by Liu *et al.*, evidently different from ours). In addition, we note with interest that the GO studied by Cao *et al.* (with a 20% functionalization level but a hydroxyl-rich composition) yields an elastic modulus also in agreement with our DFTB predictions for the GO model with a 20% functionalization level but an epoxide-rich composition. Therefore, we may reasonably assume that the elastic modulus of GO is mainly affected by the functionalization level, rather than by the relative proportions of different oxygen-containing functional groups. More specifically, the studies by Cao *et al.* and Liu *et al.* and this study contain the same relative amounts of *sp*^2^- versus *sp*^3^-type carbon–carbon bonding in systems with the same functionalization level, independent of the relative amounts of each functional group present. Thus, one can reasonably expect that the electronic structure of the GO backbone dominates the measured elastic properties of the material, that is, the identity of the bonded functional groups does not directly influence the aforementioned elastic properties.

## Discussion

To further elucidate the extent to which epoxide groups, unlike hydroxyl groups, enable GO to deform plastically, we compared the fracture surfaces obtained by Cao *et al.* with those obtained in our study (with a 70% functionalization level and an epoxide-rich composition). Cao *et al.* experimentally showed that the fracture surfaces of hydroxyl-rich GO tend to be brittle. DFT simulations predict that, for membranes of this composition, brittle failure occurs along a path populated by hydroxyl-functionalized carbon atoms. In contrast, our study shows that epoxide-rich GO fails in a ductile manner. Our simulations suggest that crack propagation is hindered due to energy dissipation through epoxide-to-ether transformations. Thus, one can reasonably conclude that the presence of epoxide groups enables GO to exhibit plastic behaviour.

Analysing the stress at the onset of plasticity allows us to further relate the material strength of GO with its level of functionalization. In contrast to the case of pristine graphene, which is nearly defect-free^1^, it is impossible to define an ‘intrinsic material strength' for GO. Instead, we used the term ‘activation stress' to describe the onset of the plastic deformation of GO, which is defined as the stress value at the membrane centre when the sample is at the plastic onset point, the last data point where the material behaves linear elastically. Since this is the first point in the stress–strain curve where plastic behaviour begins, the activation stress is analogous to the yield stress in metals. Thus, using contact analysis in the linear elastic regime^22^, the activation stress is given by


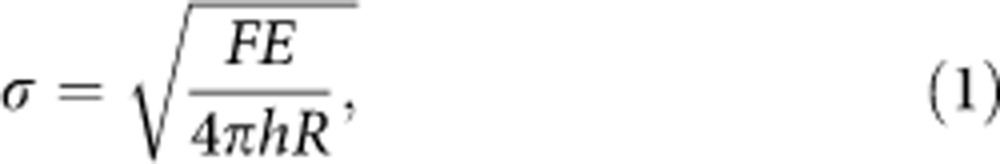


where *F* is the force at the plastic onset point and *R*=25 nm is the tip radius of the AFM probe. The experimentally determined activation stress (see Supplementary Notes) of a suspended monolayer GO is thus 5.3±1.2 GPa (or 4.0±0.9 N m^−1^), consistent with the mechanical stress applied in our DFTB simulation at the point where epoxide-to-ether transformations were activated for a GO nanosheet with *ϕ*=0.7. Given this good agreement, further equibiaxial tension simulations on GO samples with varying functionalization levels (Supplementary Fig. 6) then allow us to construct a relationship between the activation stress for the epoxide-to-ether functional group transformation and the material strength of these samples. In particular, the difference between the activation and ultimate stresses can now be used to indicate the extent of GO plasticity. As shown in Fig. 3b, while the predicted ultimate stress for GO decreases monotonically with increasing *ϕ*, the activation stress decreases up to *ϕ*=0.7 and then increases. This behaviour suggests that while the level of plasticity in GO can be increased by increasing its propensity to undergo epoxide-to-ether transformations, its effect is countered by the loss of material heterogeneity for systems with *ϕ*>0.7. Beyond this level of functionalization, further oxidization leads to the removal of graphitic domains (that is, loss of heterogeneity) so that higher strain energies are required to activate mechanochemical reactions, and, thus, loss of plasticity. This trend may also explain why this epoxide-to-ether transformation induced plasticity was not observed in previous experimental and theoretical studies of GO with either low functionalization levels or low epoxide populations^6^,^12^,^15^; sufficient functionalization levels and adequate epoxide populations are both needed for GO plasticity to become apparent.

In summary, we have established a molecular-level understanding of the unusual plasticity and defect-tolerant properties of suspended GO single layers through a synergistic combination of theoretical and experimental investigation. A novel epoxide-to-ether transformation was found to be responsible for the plasticity and ductility of GO as observed in AFM membrane deflection experiments. In contrast to the thermodynamically favourable C–O bond-cleavage pathway for epoxide ring-opening in a molecular system, the mechanically actuated ring-opening reaction of epoxides supported on the 2D basal plane of GO actually proceeds through an alternative C–C bond-cleavage pathway. As an example of a rare, if not unprecedented, bond-selective chemical transformation achieved by mechanical activation, this reaction could be used to tune the mechanical properties of GO sheets by transforming epoxide groups to more stable ether groups. We are confident that this mechanochemical approach to studying the chemistry of graphene and its derivatives will stimulate the exploration of covalent bond-selective chemistry in 2D materials, beyond that offered by proteins and oligonucleotides.

## Methods

### Materials and instrumentation

Unless otherwise stated, all reagents were used as received. Graphite powder (grade 2139) was purchased from Asbury Carbons (Asbury, NJ). Sodium nitrate, potassium permanganate, absolute ethanol, concentrated hydrochloric acid and *n*-butylamine (99.5%) were purchased from Sigma-Aldrich Co. LLC (Milwaukee, WI). Concentrated sulfuric acid, ether and methanol were purchased from VWR International LLC (Radnor, PA). Phosphoric acid (85 wt%), was purchased from Mallinckrodt Baker, Inc. (Phillipsburg, NJ). Hydrogen peroxide (30 wt% in water) was purchased from Sigma-Aldrich Co. LLC (Milwaukee, WI) and refrigerated during storage. Ultrapure deionized water (resistivity 18.2 MΩ cm) was obtained from a Milli-Q Biocel A10 system (Millipore Inc., Billerica, MA). Silicon wafers (Item # 785, 100 mm diameter, p-type, B-doped, single side polished) and silicon wafers with a 500-nm-thick thermal oxide layer (100-mm diameter, N/Phos-doped, single side polished) were purchased from University Wafer, Inc. (Boston, MA).

XPS was performed in the KECK-II/NUANCE facility at NU using a Thermo Scientific ESCALAB 250Xi (Al Kα radiation, *hν*=1,486.6 eV) (Thermo Fisher Scientific Inc., West Palm Beach, FL) equipped with an electron flood gun. XPS data were analysed using Thermo Scientific Avantage Data System software (version 5.923), and a SMART background was subtracted before peak deconvolution and integration. Scanning electron microscopy (SEM) images were taken using a FEI NovaNano 600 scanning electron microscope (FEI, Hillsboro, OR). Carbon, hydrogen and nitrogen (CHN) elemental analysis by combustion and oxygen elemental analysis by pyrolysis were performed by Micro Analysis, Inc (Wilmington, DE), with samples dried at 80 °C under vacuum for 4 h. Water content was measured by Karl Fischer titration using a C20 Compact Karl Fischer Coulometer (Mettler-Toledo International Inc., Columbus, OH) on films dried at 80 °C under vacuum for 4 h, and bath-sonicated for 5 min in dry MeOH in a sealed vial. Water contact angles were measured using a VCA Optima contact angle instrument (AST Products, Inc., Billerica, MA) by dropping 4 μl of ultrapure deionized water onto the substrate, with measurements taken at three different locations on each substrate.

### Synthesis of graphene oxide

Graphite oxide was prepared using a modified Hummer's method^7^. Briefly, a 9:1 v/v mixture of concentrated H_2_SO_4_ (360 ml):H_3_PO_4_ (40 ml) was added to a mixture of graphite (3 g) and potassium permanganate (18 g). The reaction mixture was heated to 50 °C and stirred for 12 h. The mixture was cooled to room temperature and poured over ice (∼400 ml). Then, H_2_O_2_ (8 ml of a 30 wt% solution) was added until the solution turned bright yellow. The resulting graphite oxide was filtered through a 250 μm US Standard testing sieve (VWR International LLC, Radnor, PA) and centrifuged (8,228 r.c.f. for 1 h) in a model 5804R centrifuge (Eppendorf, Inc., Westbury, NY) with the supernatant decanted away. The remaining solid was then washed with ultrapure deionized water (200 ml), HCl (200 ml of a 30 wt% solution) and EtOH (2 × 200 ml). For each wash, the mixture was filtered through the sieve and then centrifuged (8,228 r.c.f. for 1 h) with the supernatant decanted away. The remaining material was coagulated with ether (200 ml) and filtered over a polytetrafluoroethylene (PTFE) membrane (Omnipore, 5-μm pore size, Millipore Inc., Billerica, MA) overnight. The GO filter cake was then dispersed in ultrapure deionized water, with the dispersion stirred overnight. Any residual unexfoliated graphite oxide was removed by centrifuging at 8,228 r.c.f. for 5 min (2 × ) with the precipitate discarded. The final dispersion contained ∼1 mg ml^−1^ of GO, with a C:O ratio determined by elemental analysis to be 1.13. Accounting for a water content of 14.53% results in a C:O ratio of 1.57. GO films for XPS analysis were prepared by drop-casting GO solution onto silicon wafers with a thermal oxide layer, followed by drying under ambient conditions.

### Preparation of amine-modified graphene oxide

Suspended GO monolayers were deposited on patterned Si substrates by the Langmuir–Blodgett technique (see procedure below). The substrates were then placed next to three drops of *n*-butylamine on a glass slide inside of a closed petri dish and left overnight. XPS characterization of these A-GO samples (see Supplementary Fig. 7 and Supplementary Notes for details) was carried out after membrane deflection experiments were performed.

### Preparation of Si substrates with microwells

Si substrates containing arrays of microwells with 1.76-μm diameter and 4-μm depth were fabricated using a combination of photolithography and deep reactive-ion etching (DRIE). A 1.2-μm-thick photoresist layer (S1813 positive photoresist manufactured by Dow Electronic Materials Microposit, catalog number: DEM-10018348, Capitol Scientific, Inc., Austin, TX) was spin-coated onto the Si wafer at 4,000 r.p.m. using a spin coater (Cee 200X, Brewer Science, Inc., Rolla, MO). Following a 1-min soft bake at 100 °C on a hot plate, the wafer was exposed to ultraviolet light (365 nm, 18 mW cm^−2^) for 4 s on the Mask Aligner instrument (Suss MABA6, SÜSS MicroTec AG, Garching, Germany). After exposure, the wafer was developed in a MF 319 developer (manufactured by Dow Electronic Materials Microposit, catalog number: DEM-10018042, Capitol Scientific, Inc, Austin, TX) for 60 s. Spin rinsing was carried out with ultrapure deionized water (200 ml) for 30 s at ∼300 r.p.m., followed by a 60-s spin dry at 3,000 r.p.m.

The resulting photoresist-masked silicon wafer was then subjected to microwell etching using a DRIE machine (STS LpX Pegasus, SPTS Technologies Ltd, San Jose, CA). After etching, the remaining photoresist was removed using acetone, and the wafer was cleaned using isopropanol and ultrapure deionized water. This wafer was then cleaved into smaller substrates to be used in the Langmuir–Blodgett deposition and subsequent membrane deflection experiments.

Before Langmuir–Blodgett deposition, the substrates were cleaned using the following procedure: (1) submerged in 2 ml of a 3:1 v/v mixture of concentrated H_2_SO_4_:30 wt% H_2_O_2_ and heated in a Biotage (Uppsala, Sweden) SPX microwave reactor (software version 2.3, build 6250) at 180 °C for 45 min, (2) sonicated for 10 min each in ultrapure deionized water (∼10 ml), methanol (∼10 ml) and ultrapure deionized water (∼10 ml), respectively, (3) dried under a flow of nitrogen for 1 min, and (4) treated with O_2_ plasma (5 min at 190 W and 10–15 mTorr O_2_) in a South Bay Technology, Inc. (San Clemente, CA) Model PC-2000 plasma cleaner. After this cleaning process, the substrates were left under ambient conditions and their water contact angle were monitored until the desired values were reached (∼30°, 60° or 90°) before Langmuir–Blodgett deposition (see procedure below). The water contact angle of the freshly plasma-treated substrates was close to 0°, gradually increasing over time and reaching a maximum of ∼95° one week after plasma treatment.

The yield of intact suspended GO membranes is found to be strongly dependent on the water contact angle of the substrate (Supplementary Fig. 2). SEM images show that substrates with a contact angle of <60° resulted in ruptured membranes, while substrates with a contact angle of ∼60–95° yielded intact membranes. Suspended membranes that were deposited on substrates with lower water contact angle tend to rupture frequently during the drying process presumably due to the capillary pressure and surface tension of water trapped in the wells during Langmuir–Blodgett deposition, which exerts a downward force on the membrane^23^,^24^. Substrates with higher water contact angles (that is, more hydrophobic surfaces) may reduce this effect, thus preventing membrane rupture.

### Langmuir–Blodgett assembly of GO monolayers

To prepare suspended GO monolayers for the AFM membrane deflection experiments, the Langmuir–Blodgett assembly method was employed^8^. The as-prepared aqueous GO dispersion was diluted with MeOH to a mixture of 5:1 v/v MeOH:GO dispersion. The Nima technology (Espoo, Finland) model 116 trough was cleaned with acetone, and filled with ultrapure deionized water. Generally, GO solution (300–480 μl) was spread onto the water surface dropwise at a rate of 100 μl min^−1^ using a glass syringe, forming a monolayer film on the surface. The surface pressure was monitored using a tensiometer attached to a Wilhelmy plate. The film was allowed to equilibrate for at least 20 min after spreading, and then compressed by barriers at a speed of 100 cm^2^ min^−1^. The GO monolayer was transferred near the onset of the surface pressure increase (Supplementary Fig. 3) by vertically dipping the substrate into the trough and slowly pulling it up at a rate of 2 mm min^−1^.

### Atomic force microscopy membrane deflection tests

A single-crystal diamond probe (catalog number: ART D160, K-TEK Nanotechnology, Wilsonville, OR) was used to indent at the membrane centre with an AFM (Dimension 3100, Veeco, Plainview, NY) as shown in Supplementary Fig. 4a. The stiffness of the cantilever (*k*=3.18 N m^−1^) was calibrated using a standard cantilever (CLFC-NOBO, Bruker)^1^. The tip radius of the AFM probe (*R*=25 nm) was measured by an FEI NovaNano 600 SEM as shown in Supplementary Fig. 4b. All experiments were performed at room temperature and 16% humidity inside a customized environmental chamber. A constant deflection rate of 1 μm s^−1^ was used in all tests.

For a suspended circular linear elastic membrane under a central load, the force versus deflection response can be approximated as^1^


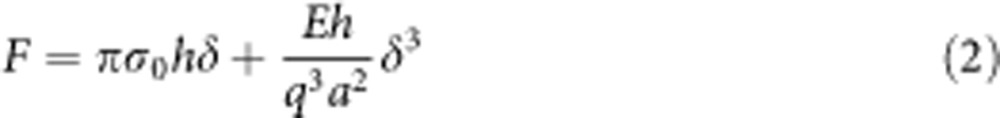


where *F* is the applied force, *δ* is the membrane centre deflection, *h* is the effective thickness of the monolayer GO membrane (taken as 0.75 nm)^21^, *σ*_0_ is the pre-stress in the membrane, *a* is the membrane diameter, *E* is the elastic modulus and *v* is the Poisson's ratio. According to DFTB calculation results (Supplementary Fig. 6), the Poisson's ratio of the GO studied here was taken as 0.2 (see Supplementary Table 3).

### Development of GO molecular models

The configurations of functional groups in GO have great impacts on the modelling results, as discussed in previous literature^12^,^13^,^14^,^15^,^16^,^17^. Thus, having physically meaningful GO models that can represent the behaviour of realistic GO sheets is important. In our work, the generation of models was carried out using a modified version of the algorithm developed by Paci *et al.*^12^. While thermodynamics favours the formation of low-energy structures over those of high energies in chemical transformations, the strongly oxidative conditions involved in the synthesis of GO are more conducive for functional groups to form stochastically (that is, kinetically driven) regardless of the relative energy associated with different oxidation pathways. In light of this, a configurational-bias Monte Carlo algorithm was modified to introduce a range of functional groups on a graphene sheet to account for both thermodynamically and kinetically driven oxidation processes^12^. The implemented algorithm comprises the following:

(1) A graphene sheet was generated with dimensions 1.988 × 2.091 nm^2^.

(2) Atoms were added in two alternating steps through a Monte Carlo addition scheme that considered all possible functionalization sites. In the first step, *N* independent and partially oxidized sheets were generated by adding two hydroxyl and four epoxide groups (one-half to each side of each sheet). Epoxide oxygen atoms were placed at a vertical distance of 1.24 Å with respect to the graphene basal planes, and at the midpoints of the lines joining two adjacent carbon atoms. Initially, hydroxyl oxygen atoms were placed at a vertical distance of 1.43 Å over carbon atoms, and associated hydrogen atoms were placed at a vertical distance of 0.95 Å over those oxygen atoms. The final, optimal C–O–H bond angles were obtained after geometry optimization.

(3) Each of the *N* sheets was subjected to geometry optimization and stress relaxation using DFTB as implemented in CP2K (http://www.cp2k.org/). This technique generates stress-free initial structures and represents the most significant modification of the algorithm proposed by Paci *et al.*^12^ Structures obtained by geometry optimization alone were found to contain compressive stresses on the order of 3 GPa, which could bias optimization results, leading to inaccurate system minima.

(4) For each of the *N* sheets, the Rosenbluth factor was calculated as given by:


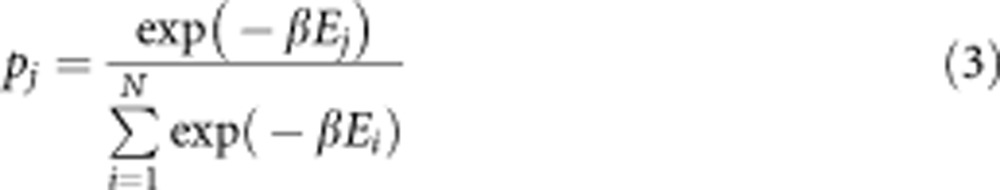


where


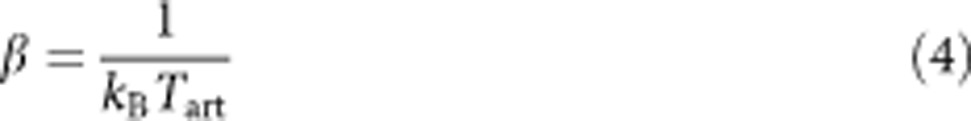


Here *p*_*j*_ is the probability of observing the *j*th sheet naturally, *E*_*j*_ is the minimized energy of the *j*th sheet, *E*_*i*_ corresponds to an energy sum over all the *N* generated GO models, *k*_B_ is Boltzmann's constant and *T*_art_ represents an artificial temperature value utilized to weight the effect of temperature in minima selection. This method, known as Rosenbluth sampling, is akin to Boltzmann distributions in statistical mechanics. The artificial temperature, *T*_art_, was chosen to be 300 K, the temperature at which membrane deflection experiments in this study were carried out.

The Rosenbluth factor for each of the *N* sheets was compared with a random number in the range [0, 1]. This process resulted in the selection of *M* structures (*M<N*) to be further oxidized, and allowed structures with relatively high energies to exist while biased for the selection of structures with relatively low energies^12^.

(5) Four additional epoxide groups were added (one-half to each side of the sheet), resulting in *N* independent sheets from each of the *M* structures. Then, geometry optimization and stress relaxation were carried out on each of the *MN* sheets and the Rosenbluth factor was calculated.

(6) GO sheets with various functionalization levels, *ϕ*=0, 0.1, 0.2, 0.36, 0.7 and 0.9, were generated by repeating steps 2–5.

This approach means the GO models generated in this study are disordered and energetically favourable (see Supplementary Fig. 8 and Supplementary Notes). All models were oxidized to a 4:1 epoxide/hydroxyl functional group ratio, based on the relative chemical composition suggested by XPS (see Supplementary Table 1). As an oxygen atom is covalently bound to two carbon atoms in an epoxide group and to one carbon atom in a hydroxyl group, the fraction of oxidized carbon atoms, *ϕ*, for each GO model is defined as:


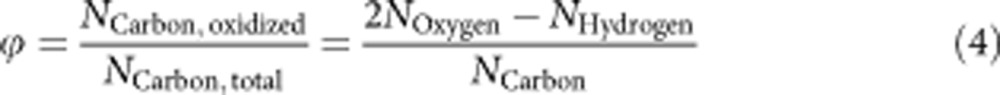


### Molecular mechanics simulation methodology

MM simulations were carried out using DFTB, a semi-empirical quantum-mechanical method^25^. This approach was chosen to balance computational efficiency and accuracy, and its performance has been demonstrated to be superior to that of classical force fields^12^,^25^. The *mio-0-1* Slater–Koster parameter set and charge self-consistency were used^26^. Charges were treated using a smooth particle-mesh Ewald summation scheme, with one grid point per Å. The Ewald convergence parameter was set to 0.35, and a cutoff radius of 10 Å for the real-space forces was used. Stresses were obtained using the virial theorem. Three types of tensile tests were carried out on graphene and GO under displacement control conditions: (i) uniaxial strain tension in the armchair direction (that is, tension was applied in the armchair direction with the boundaries in the zigzag direction fixed), (ii) uniaxial strain tension in the zigzag direction (that is, tension was applied in the zigzag direction with the boundaries in the armchair direction fixed), and (iii) equibiaxial tensile strain. Displacements of the unit-cell boundaries of the tensile direction were described according to 0.5% strain increments. Geometry optimization was carried out between each increment. Mechanical properties were extracted from MM simulations using continuum mechanics approximations for isotropic, linear elastic materials at low strains (see Supplementary Table 4, Supplementary Fig. 9 and Supplementary Notes). The results show good agreement with the experimental measurement summarized in Supplementary Fig. 10 (for a detailed discussion, please see the Supplementary Notes).

### Molecular dynamics simulation methodology

Molecular dynamics simulations were carried out based on DFTB forces. A 0.5-fs time step and the microcanonical ensemble were used. Temperature was maintained at 300 K with a Nose–Hoover thermostat and a thermostat relaxation time constant of 25 fs. One picosecond of dynamics was performed between each strain increment. Tensile strains were applied using the same procedure outlined in the molecular mechanics simulation methodology. The only molecular dynamics simulations carried out in this report correspond to equibiaxial tensile strain for the GO sheet with *ϕ*=0.7, which is representative of the material in this study. The analysis used for MM simulations was applied to extract mechanical properties from MD simulations.

## Additional information

**How to cite this article:** Wei, X. *et al.* Plasticity and ductility in graphene oxide through a mechanochemically induced damage tolerance mechanism. *Nat. Commun.* 6:8029 doi: 10.1038/ncomms9029 (2015).

## Supplementary Material

Supplementary Information Supplementary Figures 1-10, Supplementary Tables 1-4, Supplementary Note 1 and Supplementary References.
