# Erratum: Plasticity and ductility in graphene oxide through a mechanochemically induced damage tolerance mechanism

**DOI:** 10.1038/ncomms14488

**Published:** 2017-02-07

**Authors:** Xiaoding Wei, Lily Mao, Rafael A. Soler-Crespo, Jeffrey T. Paci, Jiaxing Huang, SonBinh T. Nguyen, Horacio D. Espinosa

Nature Communications 6: Article number: 8029; DOI: 10.1038/ncomms9029 (2015); Published 08 20 2015; Updated 02 07 2017

Professors SonBinh T. Nguyen and Jiaxing Huang were inadvertently omitted from the list of corresponding authors in this Article. The correct information for correspondence is: ‘Correspondence and requests for materials should be addressed to H.D.E. espinosa@northwestern.edu) or to J.H. jiaxing-huang@northwestern.edu) or to S.T.N. stn@northwestern.edu).

