# Supplementary material for: Plasticity and ductility in graphene oxide through a mechanochemically induced damage tolerance mechanism
Source: Nat Commun. 2015 Aug 20;6:8029. doi: 10.1038/ncomms9029 (PMC4560785; doi:10.1038/ncomms9029)
Supplement: Supplementary Information — Supplementary Figures 1-10, Supplementary Tables 1-4, Supplementary Note 1 and Supplementary References. [file ncomms9029-s1.pdf]

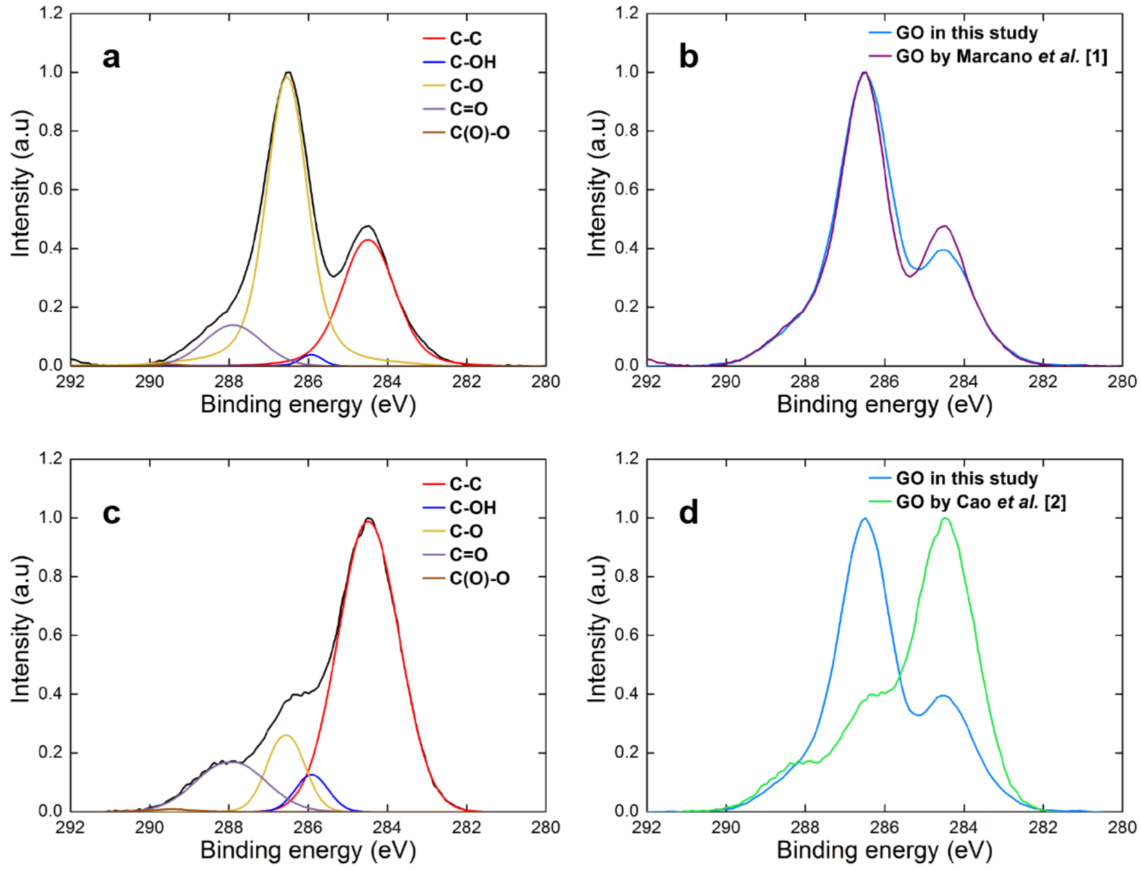

Supplementary Figure 1. Comparison of C1s XPS spectra of different GO samples. **a**, Deconvoluted C1s XPS spectrum of Marcano *et al.* [1] **b**, Overlaid C1s XPS spectra of our GO and the GO of Marcano *et al.* [1] showing the similarity between the composition of the two materials. **c**, Deconvoluted C1s XPS spectrum of Cao *et al.* [2] **d**, Overlaid C1s XPS spectra of our GO and the GO of Cao *et al.* [2] showing the clear difference between the composition of the two materials. Data shown in **(a-b)** was obtained from the authors, and data shown in **(c-d)** was digitized from the article.

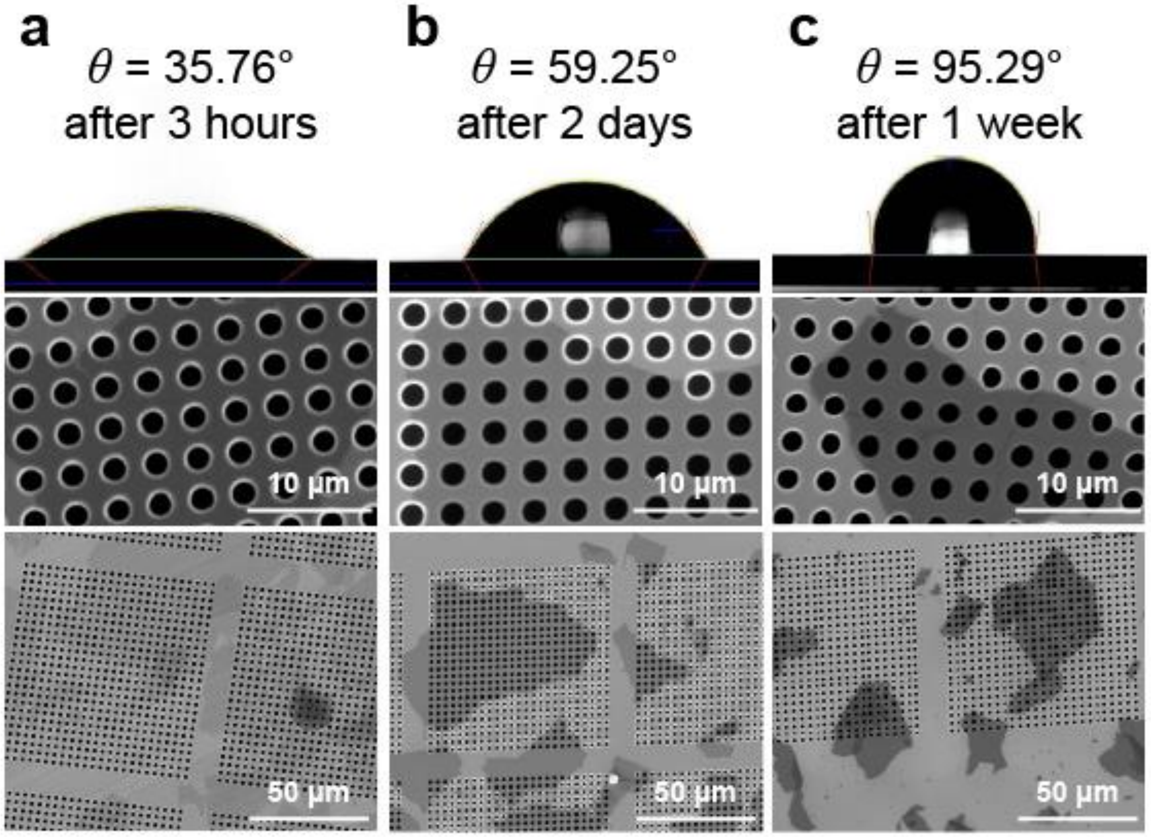

Supplementary Figure 2. Effect of *θ*, the water contact angle of the substrate, on LB deposition of suspended GO membranes. Top row: Water contact angles of bare Si substrates at various times after substrate cleaning. Middle and bottom rows: SEM images of GO monolayers deposited on the same substrates with the water contact angles shown above. Wells that contain a ruptured membrane have bright edges due to the edge effect, while those that contain intact membranes have dark edges. **a**, Deposition on a substrate with < 60° water contact angle results in ruptured membranes. **b**, Deposition on a substrate with an optimal water contact angle of ~ 60° yields intact membranes. **c**, Deposition on a substrate with > 60° water contact angle yields a lower coverage of intact but wrinkled membranes, presumably due to the hydrophobic nature of the substrate surface.

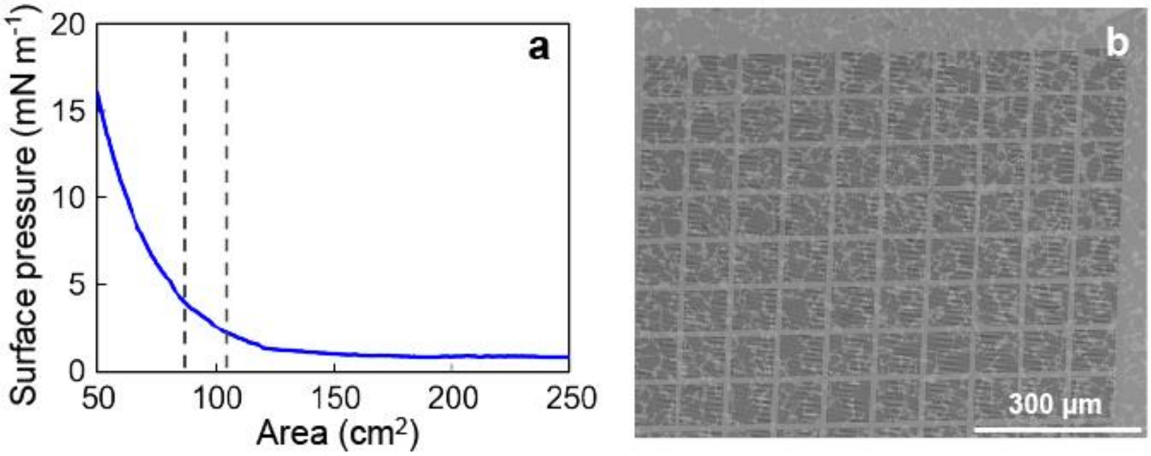

Supplementary Figure 3. LB deposition of GO monolayers. **a**, Typical surface pressure/area isotherm obtained during the LB deposition of GO monolayers. **b**, Close packed monolayers were obtained by depositing near the onset of surface pressure increase (the region indicated by the dashed lines in (**a**)).

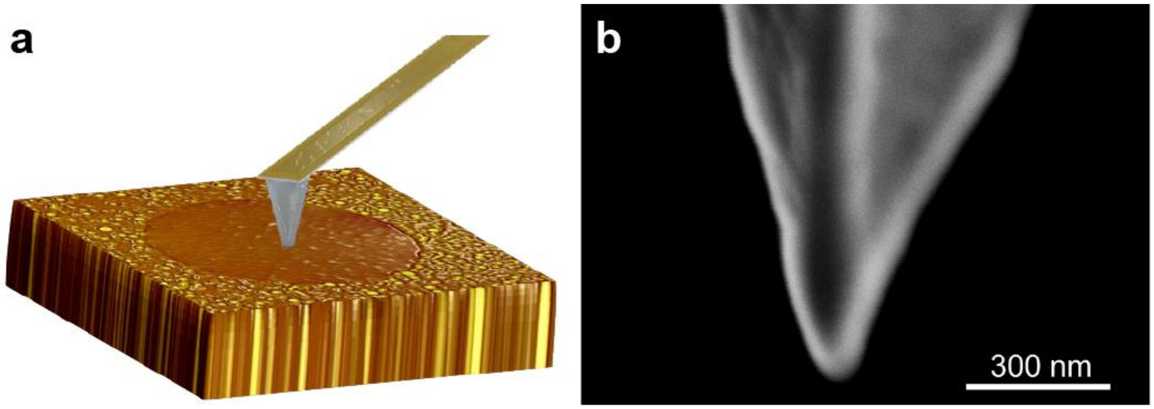

Supplementary Figure 4. AFM membrane-deflection experiment. **a**, Schematic of AFM membrane-deflection test on a suspended circular GO membrane. **b**, Scanning electron micrograph of the AFM tip.

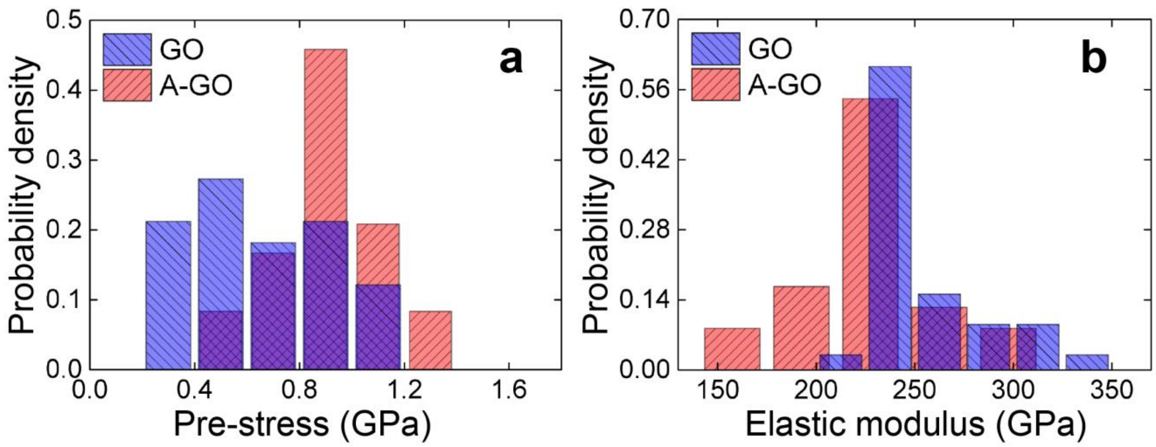

Supplementary Figure 5. Derived pre-stress and elastic modulus values. a,**b**, Histograms of pre-stress (**a**) and elastic modulus values (**b**) derived for GO and A-GO membranes.

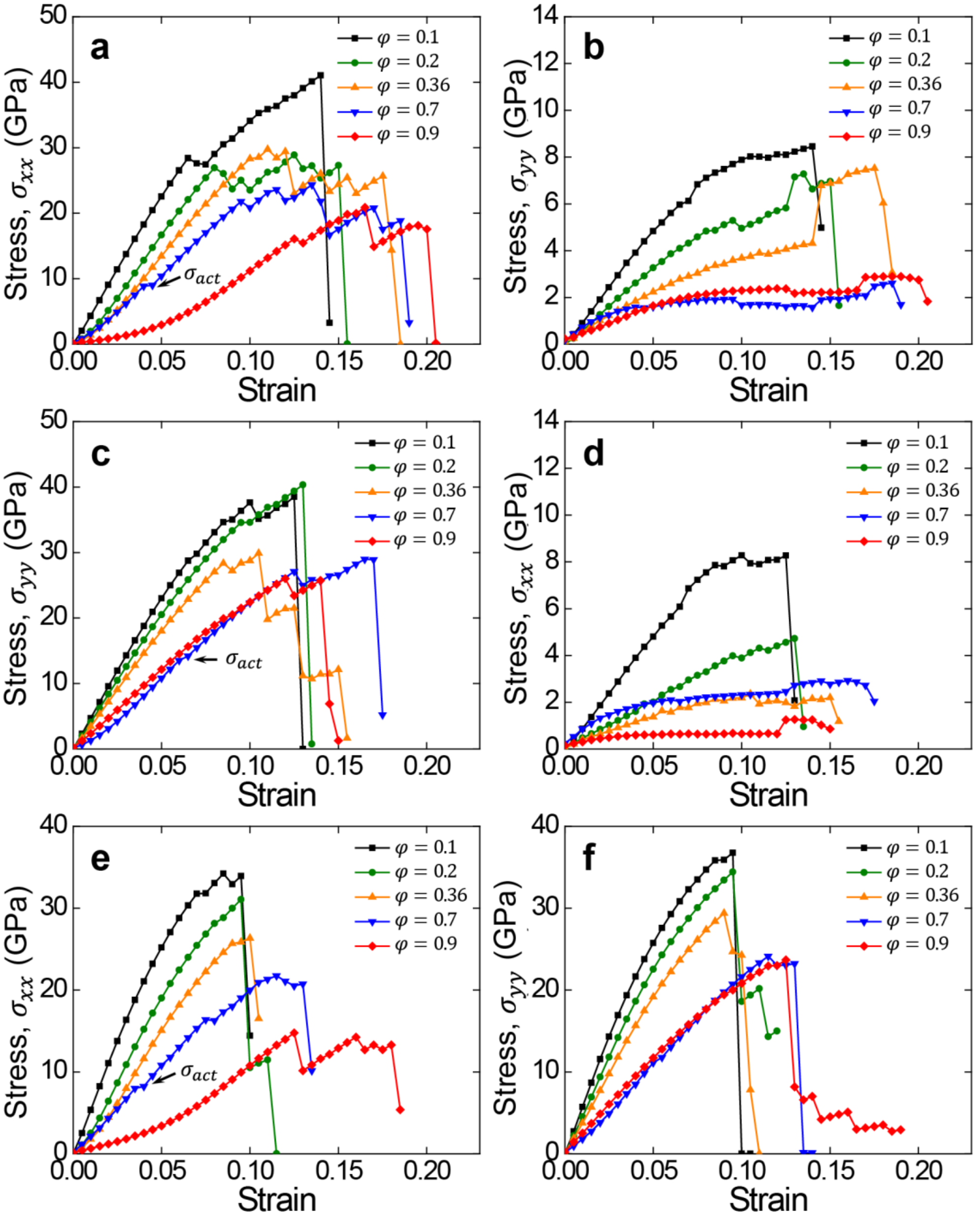

Supplementary Figure 6. Stress-strain response of GO models as a function of oxygen coverage. **a**,**b**, Stress-strain curves along the armchair (**a**) and zigzag (**b**) directions during uniaxial strain tension along the armchair direction. **c**,**d**, Stress-strain curves along the zigzag (**c**) and armchair (**d**) directions during uniaxial strain tension along the zigzag direction. **e**,**f**, Stress-strain curves along the armchair (**e**) and zigzag (**f**) directions during equibiaxial tensile strain. Marked by arrows are the activation stresses when the first epoxide-to-ether transformation occurs in 70% functionalized GO under each constraint.

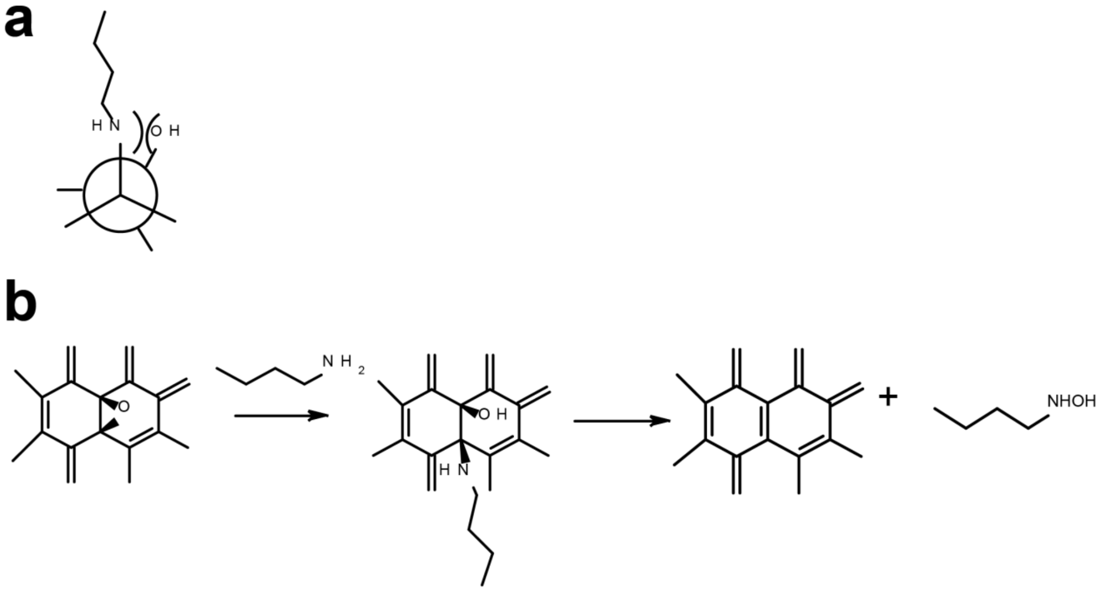

Supplementary Figure 7. Instability of vicinal amine and hydroxyl groups with *syn* configuration. **a**, Newman projection of the vicinal amine and hydroxyl groups, illustrating the Pitzer strain. **b**, Reaction scheme of *n*-butylamine functionalization of GO, showing the elimination of vicinal amine and hydroxyl groups with a *syn* configuration.

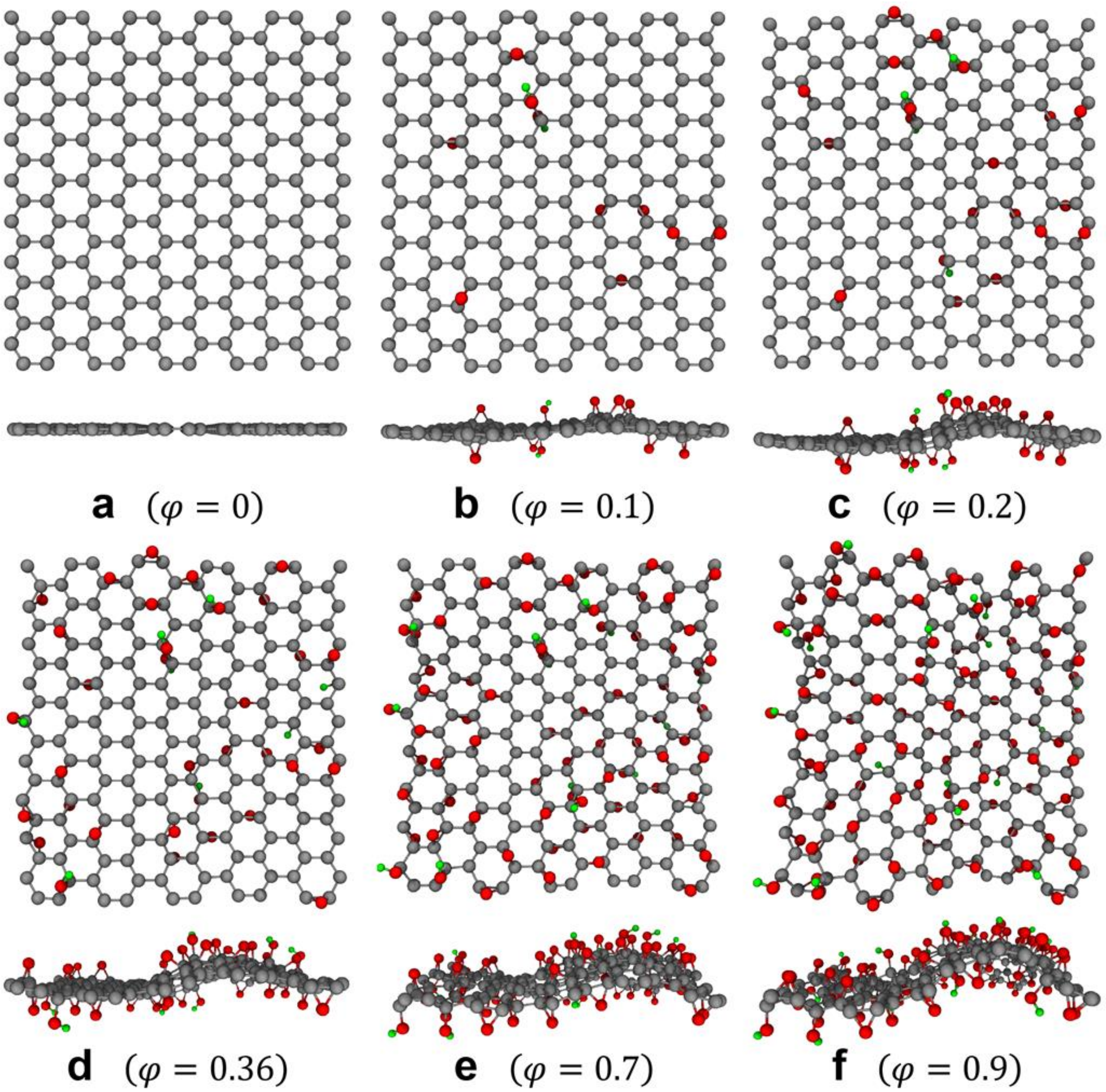

Supplementary Figure 8. Top and side views of our computationally generated models for graphene and GO, as shown along the armchair direction. a, Graphene (with 0% oxygen coverage, *i.e.*, $\varphi = 0$). **b-f**, GO with 10 ($\varphi = 0.1$, (**b**)), 20 ($\varphi = 0.2$, (**c**)), 36 ($\varphi = 0.36$, (**d**)), 70 ($\varphi = 0.7$, (**e**)), and 90 ($\varphi = 0.9$, (**f**)) % oxygen coverage. Gray, red, and green beads represent carbon, oxygen, and hydrogen atoms, respectively.

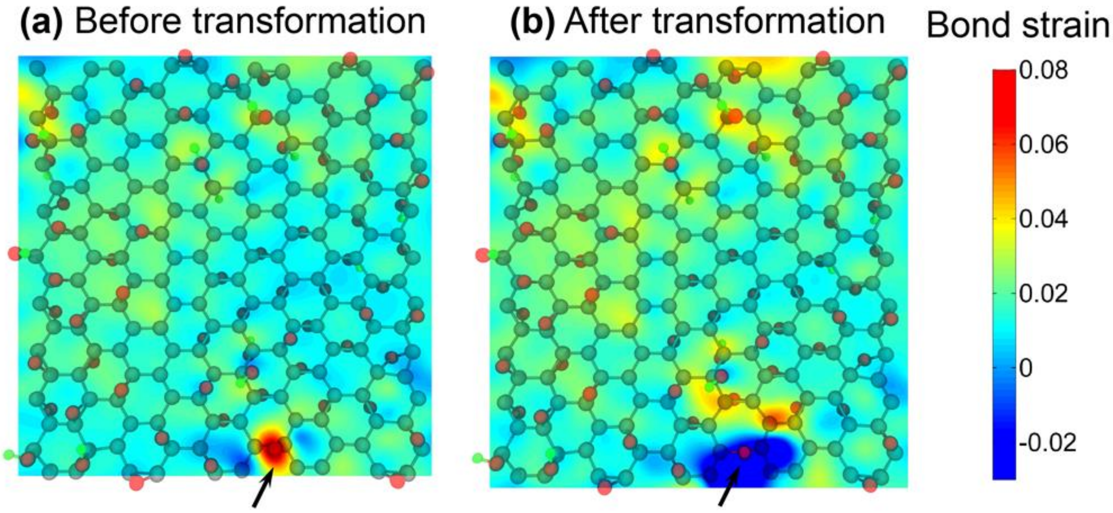

Supplementary Figure 9. Local C-C bond strain fields for GO with φ = 0.70 before (a) and after (b) the first epoxide-to-ether transformation. Arrows highlight the location where the first epoxide-to-ether transformation occurs. Gray, red, and green beads represent carbon, oxygen, and hydrogen atoms, respectively.

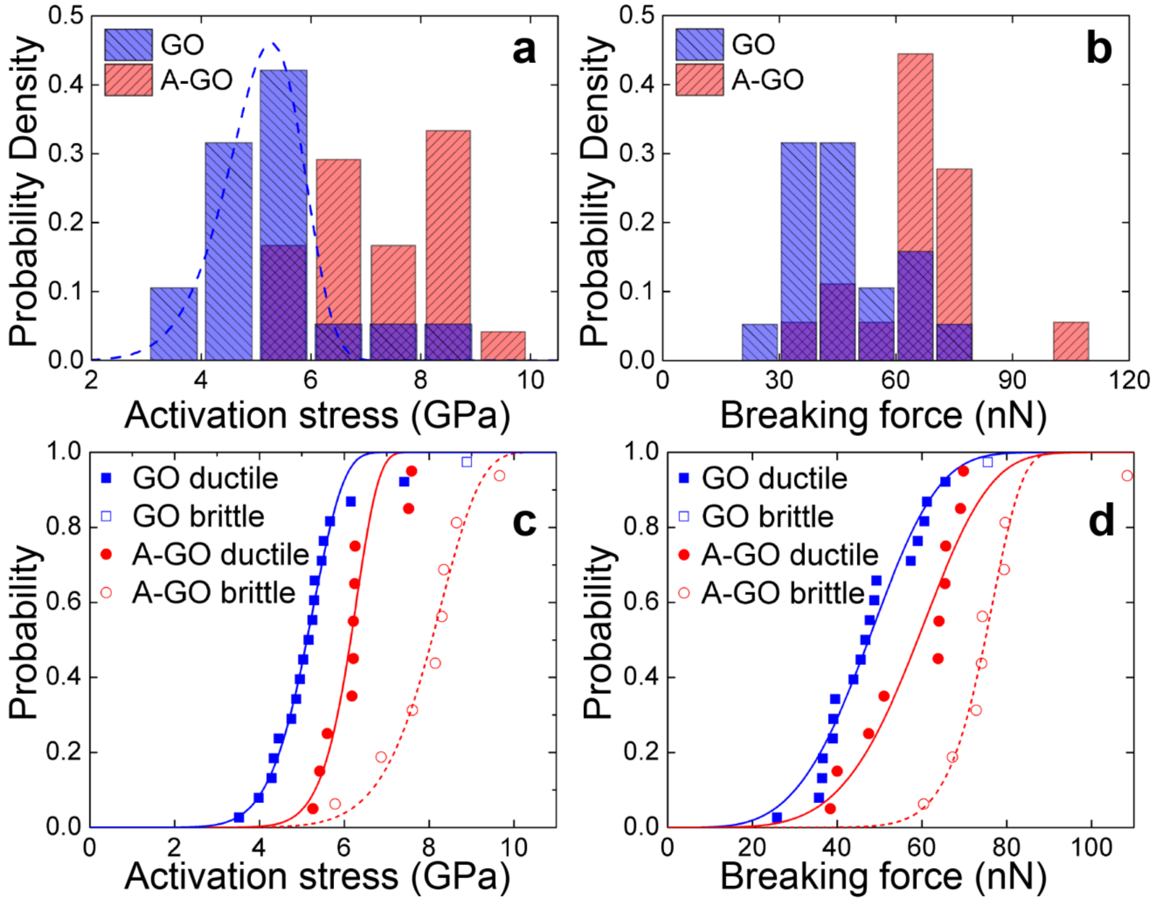

Supplementary Figure 10. Analysis of activation stress and breaking force. a, Histograms of activation stress values of GO and A-GO monolayers. Dashed line indicates the fitted Weibull distribution for GO. **b**, Histograms of the breaking force of GO and A-GO. **c**,**d**, Weibull analysis of activation stress (**c**) and breaking force values (**d**) for GO and A-GO specimens that showed ductile (solid circles) and brittle (open circles) failure, respectively.

Supplementary Table 1. Tabulated XPS peak locations and intensities for GO and A-GO [3].

Graphene oxide (GO)			Amine Graphene Oxide (A-GO)		
	BE (eV)	Relative Area		BE (eV)	Relative Area
C-C	284.46	27.4%	C-C	284.54	65.8%
C-OH	285.90	2.3%	C-OH	285.95	5.8%
C-O	286.52	60.6%	C-O	286.69	7.2%
C=O	287.88	9.5%	C=O	288.18	6.3%
C(O)O	289.42	0.3%	C(O)O	—	—
C-N	—	—	C-N	286.48	4.7%
C(O)-N	—	—	C(O)-N	287.43	10.4%

Supplementary Table 2. Tabulated XPS peak locations and intensities for GO from previous studies [1, 2].

	Marcano *et al.* GO [1]	
	BE (eV)	Relative Area
C-C	284.46	30.5%
C-OH	285.90	1.7%
C-O	286.52	57.4%
C=O	287.88	10.1%
C(O)O	289.42	0.3%

	Cao *et al.* GO [2]	
	BE (eV)	Relative Area
C-C	284.46	69.8%
C-OH	285.90	4.8%
C-O	286.52	11.3%
C=O	287.88	13.7%
C(O)O	289.42	0.5%

Supplementary Table 3. Mechanical properties derived from the MM calculations on graphene and GO with various φ. Uniaxial strain tension tests yielded the elastic modulus E, Poisson's ratio v, maximum stress $\sigma^{\max}$, and plastic strain ε^p (subscripts A and Z represent values in the armchair and zigzag directions, respectively). Additionally, σ^{act} is the activation stress in the armchair direction under equibiaxial tension. All values were calculated assuming a GO thickness of h = 0.75 nm for comparison. Values for graphene (in parentheses) assume h = 0.34 nm.

φ	E_A [GPa]	E_Z [GPa]	v_A	v_Z	$\sigma_A^{\max}$ [GPa]	σ^{act} [GPa]	$\sigma_Z^{\max}$ [GPa]	ε_A^p [%]
0	538.1 (1187.0)	541.6 (1194.8)	0.23	0.23	53.8 (118.6)	N/A	55.0 (121.3)	N/A
0.1	447.1	458.7	0.22	0.21	41.1	31.8	38.5	1.0
0.2	377.4	421.2	0.17	0.09	28.9	31.1	40.3	2.0
0.36	331.6	368.1	0.13	0.10	29.7	25.7	29.9	2.5
0.7	257.6	257.3	0.22	0.24	24.3	8.0	29.0	3.5
0.9	191.6	247.1	0.17	0.07	20.9	14.8	26.0	1.0

Supplementary Table 4. Weibull analysis results for GO and A-GO activation stresses and breaking forces.

		Activation Stress		Breaking Force	
		Scale Factor (GPa)	Shape Factor	Scale Factor (nN)	Shape Factor
GO	Total	5.4	8.3	51.8	4.1
	Ductile	5.4	8.3	51.8	4.1
	Brittle	N/A	N/A	N/A	N/A
A-GO	Total	7.5	5.4	70.7	6.8
	Ductile	6.3	12.9	63.5	4.9
	Brittle	8.4	9.7	77.2	11.8

Supplementary Note 1

XPS analysis of graphene oxide (GO) and amine-modified graphene oxide (A-GO)

XPS is a powerful technique for the characterization of the surface chemical composition of nanomaterials, and has been extensively used to study functional groups in GO [4, 5]. While XPS can detect these functional groups with high accuracy, the resulting C1s spectrum consists of several overlapping peaks corresponding to the different types of carbon atoms present. To accurately quantify the amount of each functional group, the C1s XPS spectrum must be carefully deconvoluted using the correct binding energies and number of peaks. Among previously reported studies, variation in the binding energy assigned to each functional group is presumably due to the heterogeneous chemical structure of GO. Whereas the structural model of GO includes five types of functional groups, some researchers may choose to assign only four peaks to simplify deconvolution. For example, a common practice is to fit the epoxide and hydroxyl groups as a single peak, rather than as two separate peaks. However, this does not imply that XPS cannot distinguish between epoxide and hydroxyl groups. Although these two groups are expected to have similar binding energies, the epoxide group (C-O) can exhibit a larger chemical shift (relative to the C-C group) into the emission range of the carbonyl group (C=O) [4, 6]. Indeed, several previous works separately deconvoluted and quantified epoxide and hydroxyl groups [6-8].

Analysis of XPS spectra

The C1s XPS spectrum of GO was deconvoluted into five peaks corresponding to the functional groups and binding energies (BEs) listed in Supplementary Table 1. Binding energies were based on previously reported values [3] and are in good agreement with the literature [1, 4, 6-8]. All spectra were calibrated by assigning the binding energy of the graphitic peak (C-C) at 284.46 eV. The C-C binding energy is normally assigned at 284.5-285.0 eV, with chemical shifts of +1.5, +2.5, and +4.0 eV typically assigned to the functional groups of C-OH, C=O and C(O)-O [9]. We fit the epoxide and hydroxyl groups separately, assigning the peak at 285.90 eV to the C-OH group, and the peak at 286.52 eV to the C-O group. The peaks at 287.88 eV and 289.42 eV were attributed to the C=O and C(O)-O group, respectively. Based on this analysis, the percentage of graphitic carbon atoms (C-C) in our unmodified GO is 27.4%, while the oxidized carbon atoms consist mainly of epoxides (C-O, 60.6%).

The C1s XPS spectrum of A-GO was deconvoluted into seven peaks corresponding to the five functional groups of GO and two additional groups arising from the amine modification (Supplementary Table 1). Binding energies were based on

previously reported values for amine-modified GO [3]. In contrast to the unmodified GO, the percentage of graphitic carbon atoms in A-GO is 65.8%, and that of C-O carbon atoms is 7.2%, suggesting the removal of epoxide groups and reduction of GO after amine functionalization. As noted in the main text, the removal of epoxide groups (the source of ductility in GO) is expected to lead to brittle failure of GO in membrane-deflection tests. However, both brittle and ductile failure modes for A-GO were observed, which can be attributed to the remaining epoxide groups that were not ring-opened.

Additional peaks in the deconvoluted C1s XPS spectrum of A-GO corresponding to amine (C-N, 4.7%) and amide (C(O)-N, 10.4%) carbon atoms are further evidence of the successful amine modification of GO (Fig. 1b). The N1s XPS spectrum of A-GO contains a peak (Fig. 1b), indicating the presence of nitrogen in A-GO. However, the weak intensity of this peak suggests low nitrogen loading, which is consistent with the low percentage of C-N and C(O)-N carbon atoms in the C1s XPS spectrum of A-GO (Fig. 1b and Supplementary Table 1). The low nitrogen content of A-GO can be explained by the spontaneous elimination of vicinal amine and hydroxyl groups formed during the ring-opening of epoxides by *n*-butylamine. We propose that these vicinal groups possess a *syn* configuration as one face of the GO single layer is anchored to the substrate and is thus inaccessible to amine functionalization. This is analogous to the case of vicinal *syn*-diols, which simulations predict would spontaneously detach from the GO surface [10]. Vicinal amine and hydroxyl groups may be similarly unstable due to the angle strain of tetrahedral carbons in a planar structure and Pitzer strain (Supplementary Fig. 7a) and can react further to result in loss of N-*n*-butyl-hydroxylamine and reduction of the carbon backbone (Supplementary Fig. 7b). This elimination of vicinal amine and hydroxyl groups explains the low percentage of C-N and C(O)-N carbon atoms, as well as the small increase (3.5%) in the percentage of C-OH carbon atoms and the reduction of GO after amine modification.

Comparisons of our GO XPS spectra with previously reported literature

To further confirm the epoxide-rich composition of our GO sample, we compared our C1s XPS spectrum to those of previously reported materials with well-characterized composition: highly oxidized GO with predominantly epoxide groups (synthesized by the same method we used)[1], and GO with low oxidation level and predominantly hydroxyl groups [2]. We expected our spectrum to match closely with the epoxide-rich GO and to differ dramatically from the epoxide-poor GO. As stated previously, there is variation in the reported binding energies of GO functional groups and in the number of fitted peaks used by researchers. Therefore, to make a valid comparison between all three spectra and prevent misinterpretation, the three spectra were analyzed using the same method (see

above), with the peaks assigned to the same binding energies (Supplementary Table 2 and Supplementary Fig. 1).

We obtained the raw data of the C1s XPS spectrum reported by Marcano *et al.* [1]. Deconvolution yielded an oxidation level of ~69%, in good agreement with their previously reported value and the functionalization level of our GO. Similar to our sample, the oxidized carbons consist mainly of epoxides (C-O, 57.4%). Overlaying the C1s XPS spectra of our GO and the GO made by Marcano *et al.* [1] further highlights the similarity between the two materials (Supplementary Fig. 1b). These results confirm that the composition of our material closely matches with previous studies. Marcano *et al.*[1] also characterized their sample with solid state nuclear magnetic resonance (ssNMR) spectroscopy and found that the epoxide peak was more intense than the hydroxyl peak, suggesting that epoxide groups are the predominant functional group. This validates the XPS results and given that the composition of our material is very similar (based on XPS results), this suggests that our GO is indeed epoxide-rich.

We were unable to obtain original data from Cao *et al.* [2] and instead analyzed the digitized spectrum of their C1s XPS. The spectrum was digitized using ImageJ and imported into the Avantage software. Deconvolution yielded an oxidation level of ~70%, which is similar to their reported value of 71.4%. The functional group distribution we obtained differs from what was reported by Cao *et al.* [2] presumably because we fit the epoxide and hydroxyl peaks separately. Nevertheless, we found the percentage of epoxide groups (11.3%) to be significantly lower than that of our GO. In their spectrum, it is evident that the intensity of the peak corresponding to oxidized carbons is much lower than the intensity of the peak corresponding to graphitic carbons (the opposite is true for our GO) (Supplementary Fig. 1d). This clearly shows that the composition of their GO is very different from that of our GO, and proves that an epoxide-rich composition is necessary for plasticity to be observed in GO single layers as noted in the main text.

Validation of GO model generation algorithm

Supplementary Fig. 8 shows snapshots of the top and side views for the six models generated in this study. Models with φ = 0, 0.1, 0.2, 0.36, 0.7, and 0.9 were generated to represent a range of oxidation levels potentially attainable in GO sheets. Specifically, the model with 70% oxygen coverage is consistent with the analysis reported by Marcano *et al.* (69% oxidized C) [1]. After the sheets were geometry-optimized, and the residual stresses eliminated, the microstructures of the models were quite comparable to the Lerf-Klinowski model of GO [11, 12], where hydroxyl and epoxides were the dominant functionalities. The placement and directionality of functional groups

in our model is consistent with various reports in the literature [13-17]. Specifically, we capture two reported observations. First, hydroxyl groups that appear on the same side of the basal plane are para-positioned (*i.e.*, on the opposite sides of the same hexagonal ring). On the other hand, hydroxyl groups on opposite sides of the basal plane are either meta- or ortho-positioned (*i.e.*, the second hydroxyl group is present in any of the carbon atoms that is not opposite to that bearing the first hydroxyl group). For $\varphi \geq 0.70$, a small number of carbonyl, oxetane, and ether groups were also observed, consistent with the reports of Erickson *et al.* [12]. Also, we note that while Erickson *et al.* found evidence for the formation of islands of functional groups in sheets with $\varphi \approx 0.80$, we did not include this "islanding" phenomenon in our models, as the scale on which this is thought to occur is too large to be captured by our calculations [12]. Second, we observe that sheet waviness increases in our models with increasing oxygen coverage, as reported in experiments and computations [18-21].

Interpretation of simulation results

Supplementary Fig. 6 shows a summary of the computational results obtained from MM simulations for the GO models with different oxygen coverages generated in this study. First, we were interested in extracting linear elastic properties, which can be estimated from knowledge of material elastic constants [22]. If a material is isotropic linear elastic, the following relationships hold:

$$E = \frac{C_{11}^2 - C_{12}^2}{C_{11}} \tag{1}$$

$$\nu = \frac{C_{12}}{C_{11}} \tag{2}$$

where E is the elastic modulus of the system, ν is the Poisson's ratio of the system and C_{11} and C_{12} are elastic constants.

From the uniaxial strain tension results in the armchair direction (Supplementary Figs. 6a and b), a set of elastic constants, C_{11} and C_{12}, can be determined through least-squares fitting the stress-strain curves at small strains. By applying the same procedure on the uniaxial strain tension results in the zigzag direction (Supplementary Figs. 6c and d), another set of C_{11} and C_{12} can be determined. The two sets of constants determine a set of elastic moduli (E_A and E_Z, subscripts A and Z represent values in the armchair and zigzag directions, respectively) and Poisson's ratios (ν_A and ν_Z), independently. The nonlinearity in the stress-strain curves at small strains (Supplementary Figs. 6a-d, especially for GO with φ = 0.9) arises mainly from increasing waviness intrinsic to the

sheets due to their highly oxidized nature. To alleviate this artifact, the elastic constants of the material were determined by fitting the segments where the tangential slopes stabilized. Furthermore, stress-strain curves were shifted using the stabilized tangential slopes so that the linear extrapolation of the curves passed through the origin.

As shown in Supplementary Table 3, the elastic modulus of GO decreases with increasing functionalization level due to the transition from stiff sp^2 bonds to sp^3 bonds. The elastic modulus in the zigzag direction is marginally greater than that in the armchair direction for most of the GO models. However, for the model with φ = 0.7, the elastic moduli derived for both directions are identical within numerical uncertainty. This justifies the isotropic linear elastic models used to analyze the experimental results (see equation (1) and (2) in the manuscript). We also find that the Poisson's ratio oscillates between 0.07 and 0.24, and does not show any trends in either the armchair or zigzag directions, possibly due to the stochastic directionality of epoxide groups added during model generation. The Poisson's ratio values for GO with φ = 0.7 are very close in both directions. Therefore, we chose ν = 0.2 as the Poisson's ratio in equation (2) in the manuscript for elastic analysis.

At large strains, the epoxide-to-ether group transformation (as discussed in the main manuscript) was identified in the calculations on all GO models, as shown in Supplementary Fig. 6. The activation stress was obtained by inspecting the molecular trajectories of the model at individual strain steps to directly identify the first epoxide-to-ether transformation. The stress level applied at the strain step prior to the first observed transformation was defined as the activation stress. The activation stress first decreases with increasing oxygen coverage and reached a minimum at φ = 0.7. However, when the system becomes more oxidized (φ = 0.9), it increases. This result suggests that the epoxide-to-ether transformation activation is affected by not only epoxide group population, but also material heterogeneity. Beyond φ = 0.70, more oxidization leads to loss of heterogeneity so that the transformation activation becomes more difficult. As a consequence of epoxide-to-ether transformations, all GO models exhibited plasticity before failure. To compare plasticity between different models, plastic strains were extracted by defining plastic strain (ε^p) as the strain caused purely by epoxide-to-ether transformations. As shown in Supplementary Table 3, the trend in plastic strain is opposite to that of the activation stress. The simultaneous minimum activation stress and maximum plastic strain at φ = 0.70 suggest that GO ductility is optimized at this specific chemical composition. Finally, the ultimate strength ($\sigma^{\max}$) monotonically decreases when φ increases as a result of more sp^3 bonds, which are weaker than sp^2 bonds. In summary, our calculations suggest that there is ample space for tuning the mechanical properties

(*i.e.*, elastic modulus, strength and ductility) of GO. Based on our results, the material studied in this report should possess *optimal* ductility.

To provide further insight into the epoxide-to-ether transformation mechanism, we extracted the local C-C bond strain information in the 70% functionalized GO model before and after the first transformation. As shown in Supplementary Fig. 9, there is a highly localized tensile strain in the C-C bond of the epoxide group prior to its transformation (Supplementary Fig. 9a). In contrast, the C-O bonds in that epoxide ring undergo much less strain. This demonstrates why the C-C bond breaks instead of the C-O bonds when an epoxide group in GO is subjected to a critical in-plane mechanical load. After the transformation (Supplementary Fig. 9b), a significant strain relaxation was observed in the same location. This local C-C bond strain analysis provides further insight into how the transformation mechanism helps arrest the nano-cracks in GO and affords the material enhanced toughness as discussed in the main text.

Analysis of AFM membrane-deflection test results

Elastic analysis

By fitting the force vs. deflection response with equation (2) in the manuscript, while keeping σ_0 and E as two free variables, both the pre-stress and elastic modulus were determined. For all tests, the experimental curves were only fitted up to the plastic onset points. To avoid artifacts, the plastic onset point was determined as the data point beyond which the least-squares fitting using equation (2) of the manuscript produces an R^2 value lower than 0.999. Elastic analysis results for GO and A-GO samples are compared in Supplementary Fig. 5.

Weibull analysis of activation stresses and breaking forces

The two-parameter Weibull probability distribution function is expressed as:

$$P = 1 - \exp\left\lfloor -\left(S / S_0\right)^m \right\rfloor \tag{3}$$

where P is the probability of material damage or failure for the material subjected to a quantity S, S_0 is the scale factor for the corresponding quantity, and m is the shape factor (or Weibull modulus) that determines the breadth of the probability distribution. A small m means a wide variation in the analyzed quantity that implies a broad range of defects in the material. A large m means either that the material failure is insensitive to the presence of defects or that there is a narrow range of defects in the material. In this study, the quantities of interest in Supplementary equation (3) are activation stress and breaking force.

Supplementary Figs. 10a and b show the distributions of activation stress and breaking force for GO and A-GO. As discussed in the manuscript, both GO and A-GO specimens exhibited ductile and brittle types of force vs. deflection curves but with different statistical distributions. All GO samples except for one showed ductile failure, presumably due to the plasticity that originated from the epoxide-to-ether group transformation as discussed in the main paper. In contrast, a significant portion (~ 44%) of the A-GO specimens failed in a brittle way, indicating the presence of a different failure mechanism. Indeed, the bi-modal distribution of the A-GO activation stress values (Supplementary Fig. 10a) suggests that Weibull analysis should be carried out on the data sets of A-GO for each failure mode to evaluate the different mechanisms. As such, individual Weibull analysis was carried out separately on the two classes of A-GO specimens that exhibited ductile and brittle modes of failure (Supplementary Table 4).

Weibull analysis of the activation stress of ten ductile A-GO membranes yields a scale factor of 6.3 GPa (shape factor = 12.9), lower than that for the eight brittle A-GO membranes (scale factor = 8.4 GPa, shape factor = 9.7) (Supplementary Fig. 10 and Supplementary Table 4). Both of these values are higher than the activation stress of all GO membranes (scale factor = 5.4 GPa, shape factor = 8.3). Weibull analysis of the breaking force values (Supplementary Fig. 10d) yields a similar trend: The scale factor for the ten ductile A-GO membranes (63.5 nN, shape factor = 4.9) is lower than that for the eight brittle A-GO membranes (77.2 nN, shape factor = 11.8) but higher than that for all GO membranes (scale factor = 51.8 nN, shape factor = 4.1). Interestingly, the shape factor of the breaking force for the ductile A-GO is very close to that for all GO membranes, suggesting the same failure mechanism governs both materials. The larger scale factors of the Weibull analyses of the breaking forces for both ductile and brittle A-GO specimens, compared to that for the GO membranes, suggest that A-GO has gained much-improved mechanical strength with only a partial loss of plasticity. However, the shape factors of the Weibull analyses of the breaking forces for both GO and A-GO are still significantly lower than that for pristine graphene (~16) [22], indicating failure is highly affected by imperfect lattices and inherent voids in the materials.

Supplementary References

1. Marcano, D.C. et al. Improved synthesis of graphene oxide. *ACS Nano* **4**, 4806-4814 (2010).
2. Cao, C., Daly, M., Singh, C.V., Sun, Y. & Filleter, T. High strength measurement of monolayer graphene oxide. *Carbon* **81**, 497-504 (2015).
3. Compton, O.C., Dikin, D.A., Putz, K.W., Brinson, L.C. & Nguyen, S.T. Electrically conductive "alkylated" graphene paper via chemical reduction of amine-functionalized graphene oxide paper. *Adv. Mater.* **22**, 892-896 (2010).
4. Yang, D. et al. Chemical analysis of graphene oxide films after heat and chemical treatments by X-ray photoelectron and Micro-Raman spectroscopy. *Carbon* **47**, 145-152 (2009).
5. Dreyer, D.R., Park, S., Bielawski, C.W. & Ruoff, R.S. The chemistry of graphene oxide. *Chem. Soc. Rev.* **39**, 228-240 (2010).
6. Pulido, A. et al. Reconstruction of the carbon sp2 network in graphene oxide by low-temperature reaction with CO. *J. Mater. Chem.* **22**, 51-56 (2012).
7. Koinuma, M. et al. Analysis of Reduced Graphene Oxides by X-ray Photoelectron Spectroscopy and Electrochemical Capacitance. *Chem. Lett.* **42**, 924-926 (2013).
8. Ganguly, A., Sharma, S., Papakonstantinou, P. & Hamilton, J. Probing the Thermal Deoxygenation of Graphene Oxide Using High-Resolution In Situ X-ray-Based Spectroscopies. *J. Phys. Chem. C* **115**, 17009-17019 (2011).
9. Yumitori, S. Correlation of C1s chemical state intensities with the O1s intensity in the XPS analysis of anodically oxidized glass-like carbon samples. *J. Mat. Sci* **35**, 139-146 (2000).
10. Lahaye, R., Jeong, H., Park, C. & Lee, Y. Density functional theory study of graphite oxide for different oxidation levels. *Phys. Rev. B* **79**, 125435 (2009).
11. Lerf, A., He, H., Forster, M. & Klinowski, J. Structure of graphite oxide revisited. *J. Phys. Chem. B* **102**, 4477-4482 (1998).
12. Erickson, K. et al. Determination of the local chemical structure of graphene oxide and reduced graphene oxide. *Adv. Mater.* **22**, 4467-4472 (2010).
13. Bagri, A. et al. Structural evolution during the reduction of chemically derived graphene oxide. *Nat. Chem.* **2**, 581-587 (2010).
14. Boukhvalov, D. & Katsnelson, M. Tuning the gap in bilayer graphene using chemical functionalization: density functional calculations. *Phys. Rev. B* **78**, 085413 (2008).
15. Yan, J.-A., Xian, L. & Chou, M. Structural and electronic properties of oxidized graphene. *Phys. Rev. Lett.* **103**, 086802 (2009).
16. Wang, L. et al. Stability of graphene oxide phases from first-principles calculations. *Phys. Rev. B.* **82**, 161406 (2010).
17. Liu, L. et al. Amorphous structural models for graphene oxides. *Carbon* **50**, 1690-1698 (2012).
18. Incze, A., Pasturel, A. & Peyla, P. Mechanical properties of graphite oxides: *Ab initio* simulations and continuum theory. *Phys. Rev. B* **70**, 212103 (2004).

19. Dikin, D.A. et al. Preparation and characterization of graphene oxide paper. *Nature* **448**, 457-460 (2007).
20. Wei, N., Lv, C. & Xu, Z. Wetting of graphene oxide: a molecular dynamics study. *Langmuir* **30**, 3572-3578 (2014).
21. Qiu, L. et al. Controllable corrugation of chemically converted graphene sheets in water and potential application for nanofiltration. *Chem. Commun.* **47**, 5810-5812 (2011).
22. Lee, C., Wei, X., Kysar, J.W. & Hone, J. Measurement of the elastic properties and intrinsic strength of monolayer graphene. *Science* **321**, 385-388 (2008).
